# New Infiltration Technique in the Treatment of the Plantar Fascia Syndrome Based on Platelet-Rich Plasma

**DOI:** 10.3390/jcm13010170

**Published:** 2023-12-28

**Authors:** Francesc Pardo-Camps, Francesc Pardo-Bosch

**Affiliations:** 1 University Clinic, Complutense University of Madrid, Plaza Ramón y Cajal, s/n, 28040 Madrid, Spain; drpardocamps@drpardocamps.com; 2 Departament of Orthopedic Surgery and Traumatology, Catalan Institute of Health, Av. Josep Laporte 2, 43204 Reus, Spain; 3 Departament of Project and Construction Engineering, Universitat Politècnica de Catalunya (BarcelonaTech), C. Jordi Girona 1-3, 08034 Barcelona, Spain

**Keywords:** platelet-rich plasma, PRP, infiltration, plantar fascia syndrome, conservative treatment

## Abstract

Pain in the attachment of the plantar fascia in the calcaneus represents 10% of all sports injuries, affects 10% of foot runners, and will affect around 20% of the world population. There is no effective conservative treatment for it. This paper justifies a new definition and name for this pathology, Plantar Fascia Syndrome (PFS), presents a methodology for its diagnosis, and presents the clinical and functional effectiveness of a new conservative treatment based on platelet-rich plasma (PRP). In total, 25 patients (from an initial sample of 260) diagnosed with recalcitrant PFS lasting for more than 12 months were treated with a single infiltration of 2 mL of PRP, according to a new technic proposed. The study was approved by the ethical committee for clinical research of the reference hospital. The patients were controlled after 15, 30, 90, and 180 days, reviewing on each occasion pain, thickness of the plantar fascia, and active extension of the ankle joint. A total of 15 days after infiltration, 85% of patients had no clinical signs requiring treatment. After 90 days of infiltration, no patients showed clinical signs. This improvement in the patients’ condition lasted for 180 days. All patients after treatment can fully resume normal activity with no pain.

## 1. Introduction 

Feet are the most distal functional units in the lower part of the human body. They are the basic mechanism that maintains the balance in the bipedal position. Feet’s movements are combined and sequential. Desynchronization in these movements leads to a degeneration of the structures, which in turn results in pain. Foot pain can be disabling, which explains why there are so many patients seeking medical and surgical help. 

One frequently painful anatomical structure of the foot is the enthesis or the site of attachment of the plantar fascia (aponeurosis) in the calcaneus. This pain occurs due to stiffness after prolonged rest. The pain originates in different pathologies that affect both the fascia and the calcaneus, as well as other adjacent structures. This injury harms 7% of the population over 65 years old who experience pain in this area of the foot [1]. In foot-racing athletes, this injury accounts for 25% of all foot injuries [2] and approximately 8 to 10% of all injuries. Despite the importance of this injury, the relief of the symptoms takes about 1 year [3], and the outcome is not always promising.

**Figure 1:**
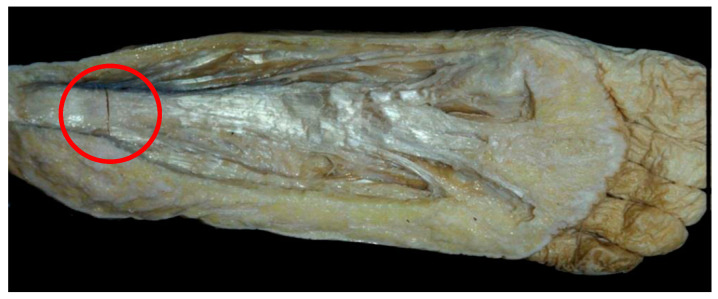
Location of the Plantar Fascia Syndrome in the hindfoot.

The pathology of the enthesis between the plantar fascia and the calcaneus (circled area in Figure 1) arising from opposing tensions of the various forces that converge at this point has been investigated for more than 100 years without a clear description of its aetiology, a well-defined diagnostic methodology, or a precise treatment protocol. To date, this pathology has been referred to as plantar fasciitis [4,5] or plantar fasciosis [6,7].

After having studied the pathological signs of this syndrome and considered its definitions, the authors have named this pathology *Plantar Fascia Syndrome* (PFS) with the following definition: “*Sharp and stabbing pain after resting the foot on the floor, located in the anterior lower face of the heel, and irradiating and/or projecting to the middle of the sole of the foot*” [8]. Pain is the primary symptom in patients with PFS and is associated on many occasions with tightness or stiffness of the plantar area, a reduction in mobility of the arch of the ankle [9], and a certain degree of progressive functional impairment. This definition is supported by Nuclear Magnetic Resonance (MRI) studies, where it is possible to detect the presence of bone oedema, subchondral lesions, and various other bone pathologies that coexist with the inflammatory pathology, sometimes chronic, of the plantar fascia itself. 

The connective tissue and the muscle-tendinous fibres of the plantar fascia have good regenerative capacity, however, depending on the extent of the pathology, assistance may be needed to avoid incomplete regeneration [10]. On the other hand, the calcaneus presents chondral injuries of difficult regeneration due to vascular deficit, which would benefit from the angiogenic growth factors (GFs) [11]. In the last decade, contributions from molecular biology have enabled the development of new techniques. These techniques include the application in the injured area of platelet-rich plasma (PRP) preparations. Its utility has been demonstrated in different fields such as bone regeneration [12,13], ophthalmologic surgery of the limbus and cornea [14,15], cardiopulmonary surgery [16], maxillofacial surgery [17,18], dentistry [19], traumatology, accelerating the healing of both muscle and tendon injuries, and in osteo-degenerative joint injuries [20,21,22,23].

Nowadays, PRP is seen as a non-industrial platelet concentrate with a platelet concentration greater than the basal count. When activated, it releases GFs with anti-inflammatory, analgesic, and regenerative properties. In daily practice, it is recognised that these effects can, in general, stimulate the tissue repair processes in both bone and soft tissues, decrease the infection rates in following treatments [24], reduce pain and inflammation, and, finally, reduce blood loss [25,26].

Considering the clear regenerative power of PRP, the good results obtained with PRP, which is considered a conservative treatment [27,28], in other musculoskeletal pathologies, and the fact that PFS is a pathology characterised by deterioration of soft tissue (fascia) and hard tissue (calcaneus), this paper aims to demonstrate the effectiveness from a clinical and functional point of view of a conservative treatment based on only one infiltration of 2 mL of platelet-rich plasma (PRP) in patients diagnosed with Plantar Fascia Syndrome (PFS) with a new injection procedure.

The enthesis between the plantar fascia and the calcaneus is a biomechanical site subject to different tractions, cutting, and twisting forces. Its dynamic balance is maintained due to its elastic properties. The biomechanical action of the enthesis requires significant energy consumption, which in turn results in wear and tear or the deterioration of the insertional fibres. Many authors believe that these fibres may be repaired and/or recovered through the external application of PRP. This application of PRP also aims to improve the osteocartilaginous tissue where the plantar fascia is inserted to perform its physiological action of traction-distraction and where other forces, such as cutting and twisting forces, converge.

## 2. Materials and Methods

### 2.1. Type of Study

**Figure 2:**
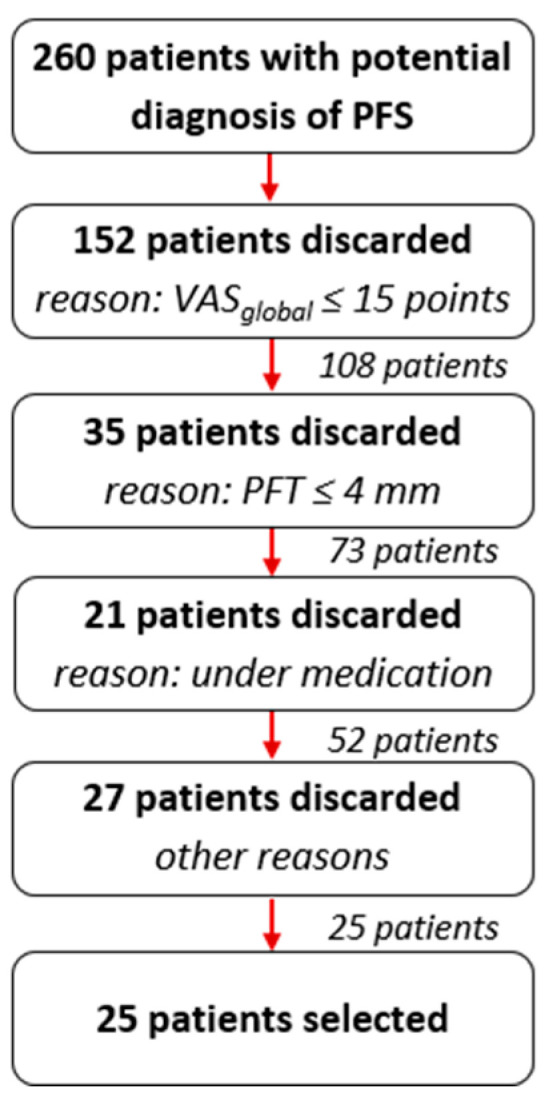
Selection scheme of the 25 patients.

The authors conducted a multicentre interventional clinical study with volunteer patients diagnosed with Grade III Plantar Fascia Syndrome (see Section 2.2) and subjected them to treatment based on a single infiltration of 2 mL of platelet-rich plasma (PRP), classified as pure platelet-rich plasma according to the platelet concentrates’ classification [29]. The device used was Proteal^®^ (Gavà, Spain), homologation reference CE 0318. All patients met the inclusion criteria: pain evaluation using the VAS_global_ equal to or greater than 15 points; ultrasound evaluation showing a proximal thickening of the plantar fascia greater than or equal to 4 mm, measured 5 mm away from the medial tuberosity of the calcaneus in the longitudinal section; pathology duration of equal to or greater than 6 months; men and women over 18 years old; willing to participate in the study after signing the informed consent. They did not meet any exclusion criterion: presence of systemic, degenerative, neurological, and sensory diseases impacting or manifesting the ankle and foot; morpho-functional alterations impacting the ankle and foot leading to significant clinical discrepancies in the lower limbs, dysmetries, or obvious clinical scoliosis; having received any medical, orthopaedic and/or nonsurgical invasive treatment for the enthesis condition between the plantar fascia and the calcaneus in the last three months; presenting any type of platelet or coagulation disorder; or having difficulties in understanding the treatment instructions. The study, carried out between May 2013 and December 2014, was approved by the ethical committee for clinical research of the ‘Hospital Clínico San Carlos’ in Madrid (internal code: 13/179-e). The study started with 260 patients with potential diagnosis of PFS, according to the primary health care doctors in the institutions where the study was conducted. After the relevant technical checks, developed by the researchers (see Figure 2), were conducted, 25 patients were selected for being treated with PRP. 

Out of the initial 260 patients, 152 were excluded as their pain assessment using VAS_global_ did not reach the required minimum of 15 points (see Section 2.2). Additionally, 35 patients were discarded as their plantar fascia thickness measured less than 4 mm. Another 21 patients were excluded due to medication (received corticosteroid infiltration or took nonsteroidal anti-inflammatory drugs). Furthermore, 9 patients were excluded for ongoing orthopaedic treatment, 3 for presenting platelet or coagulation disorders, 9 due to lack of willingness to participate in the study, and 6 for difficulties in comprehension.

### 2.2. Plantar Fascia Syndrome: Diagnosis and Classification

The primary diagnosis of PFS is basically clinical, but nowadays confirmation by means of the so-called complementary diagnosis tests is required.

Pain in the medial calcaneal tuberosity can be evaluated with the 10-point Visual Analogue Scale (VAS), where the patient is asked to score his/her level of pain, with 0 meaning ‘no pain’ and 10 meaning ‘severe pain’. The global assessment of pain (VAS_Global_) takes place in three stages or occasions, as shown in Equation 1. The first pain assessment (VAS_1_) is conducted when the patient gets out of bed, puts the affected foot on the floor, and takes his/her first steps; the second pain assessment (VAS_2_) is conducted when the patient puts the affected foot on the floor after a prolonged rest (minimum duration of 30 min); and the third pain assessment (VAS_3_) is conducted when the patient’s medial calcaneal tuberosity is touched or pressed during the clinical examination. This clinical-exploratory protocol has been designed according to different works [3,30,31].

(1) $${\mathrm{VAS}}_{\mathrm{Global}}=\overset{3}{\underset{i=1}{\sum }}{\mathrm{VAS}}_{i}$$

Based on the result of this triple assessment, each patient can be classified into three groups: Group A (VAS_Global_ ≤ 15 points), Group B (15 points < VAS_Global_ ≤ 20 points), and Group C (VAS_Global_ > 20 points).

The second element that allows the doctor to make a clear diagnosis of PFS is the thickness, in millimetres, of the average strip of the plantar fascia measured using ultrasonography (US) in the longitudinal plane at a distance of 5 mm from the medial calcaneal tuberosity. The US examination was developed by specialist doctors from the radiodiagnosis service of the medical centres in which the study was carried out. They followed the protocol outlined in the European technical guide for musculoskeletal ultrasound for the ankle and foot. The devices used were the Esaote MyLab gold 25 model and the Esaote Tecohnus MPX (Sant Just Desvern, Spain). This is an objective element that when in the hands of an expert, can be considered as pathognomonic in confirming a diagnosis of PFS. A thickness lower than 3 mm can be considered normal [32]. Other studies [33,34] consider a positive diagnosis of PFS when the thickness is higher than 4 mm. Based on these assessments, PFS can be classified into 3 groups according to plantar fascia thickness (PFT): Group 1 (PFT ≤ 3 mm), Group 2 (3 mm < PFT ≤ 4 mm), and Group 3 (PFT > 4 mm).

**Figure 3:**
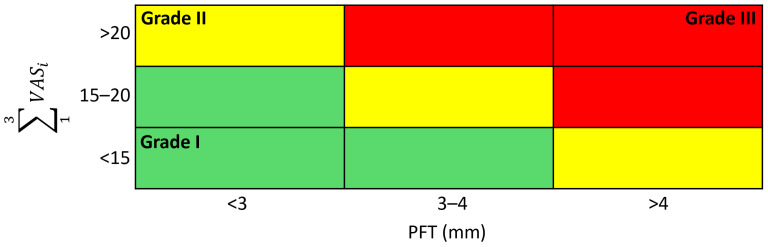
Classification of the Plantar Fascia Syndrome.

The combination of both classifications defines three grades of PFS, as shown in Figure 3, which are: Grade I (mild), Grade II (moderate), and Grade III (severe). 

### 2.3. Procedure—Infiltration Technique

This paper presents a new therapeutic technique for the treatment of PFS based on knowledge of the pathogenesis and of the anatomy of the sole of the foot and is intended to address the causes of the disease through bio-regenerative therapy using PRP infiltration. Although PRP infiltration has been reserved for Grade III patients, there is no reason why it cannot be used in patients in earlier stages or less severe forms of PFS. 

#### 2.3.1. Preparation of the Patient

The patient lies on a stretcher in a supine decubitus position with the affected member in external rotation and the knee flexed about 70°. With this movement, the foot will be in a side support position with the internal face looking upwards (the puncture for PRP injection will be performed in this internal area). The surgical field for the foot to be treated will be prepared according to the same protocol used in any other minor outpatient surgery. After the patient has been adequately prepared and with the medial face of the calcaneus fully exposed, the location of the anterior-internal calcaneal tuberosity (the point of maximum pain) will be determined by palpation or by ultrasonography. This is the reference point for the PRP infiltration. Just before the infiltration, local superficial anaesthesia will be performed by spraying the skin with chloroethyl Chemirosa^®^ (Laboratorios ERN, Barcelona, Spain), where ethyl chloride is applied until the skin begins to whiten [35]. The patient will not experience any pain when the needle is introduced. 

#### 2.3.2. PRP Administration

*PRP activation*. PRP is activated with calcium chloride 5% and according to the established protocol. The infiltration must be performed between 6 and 10 min after the PRP has been activated. 

*Infiltration point*. The infiltration should be performed at the anterior-inner edge of the calcaneus taking specific incision and/or penetration lines following the skin folds as a reference. Plantar load areas should be avoided in order to minimise the risk of injury to any vasculo-nervous structure and to reduce the risk of infection. Another reason for recommending this anatomical area is the fact that plantar skin is connected to the underlying fascia by means of many strong fibrous fascicles called retinacula cutis that act as partitions dividing the subcutaneous fat into small irregular chambers or compartments containing tiny vessels to the dermis. They also prevent movement between the skin and fascia. These chambers are larger in the rear part of the foot, below the calcaneus, where they act as miniature shock absorbers. So, if the infiltration is given in the ventral face of the calcaneus, the shock-absorbing function can be compromised and lead to heel pain syndrome [36].

**Figure 4:**
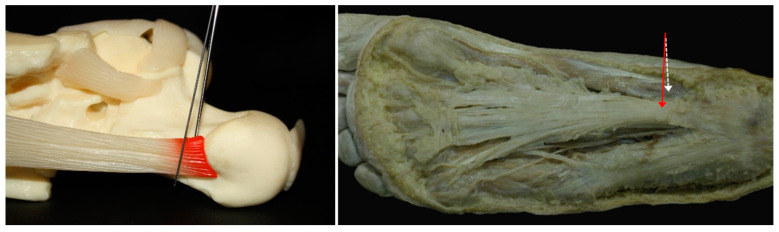
Theoretical needle insertion point for the injection of PRP.

*Technique of execution*. The needle is inserted perpendicularly to the skin (at an angle of 90°), two centimetres from the plantar plane (lower edge of the calcaneus), in the transition area between the skin of the medial face of the heel and the skin of the plantar area and according to a linear plane between 1 and 3 mm from the medial tuberosity (see Figure 4). An expert traumatologist should not need to use ultrasonography (US) to ensure the correct positioning of the needle; however, US could help new practitioners.

**Figure 5:**
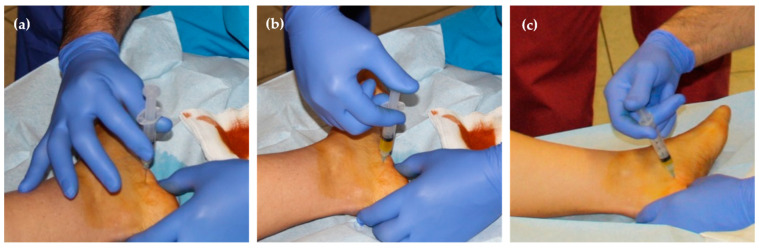
PRP administration: (**a**) plantar fascia palpation; (**b**) caudal injection; and (**c**) ventral injection.

When the puncture is performed at an angle of 90°, the needle reaches the plantar fascia so that when the therapist tries to press the plunger of the syringe there is considerable resistance (Figure 5a) and is unable to inject the PRP. For this reason, the needle must be withdrawn about 5 mm and immediately reinserted at an angle of 5° in the caudal direction so that the plasma can be injected without resistance. The first millilitre of the PRP is injected with the needle in this position (Figure 5b). Then, the needle must be withdrawn about 5 mm until it returns to a plane perpendicular to the skin and reinserted at an angle of 5° in the ventral direction so that the remaining millilitre of PRP can also be injected without resistance (Figure 5c).

The infiltration is given at an angle of 5° both in the caudal and ventral directions because that allows the PRP to be injected into two anatomical cavities big enough to receive the 2 mL. For this reason, there is no need to press hard on the plunger of the syringe (which confirms that the PRP has been injected in the right place). With this technique, PRP is applied to the whole plantar fascia inserted in the calcaneus, as well as in all the medial tuberosity, which are precisely the anatomical elements affected by the degenerative pathology requiring treatment. 

### 2.4. Data Collection

The 25 patients (see Table 1 for the demographic features of the population) selected to participate in the observational study received infiltrations in accordance with the above-described technique. 

The evolution of the most significant clinical variables was studied for a period of 6 months from Day 0 at 4 control visits (Day 15, 30, 90, and 180). The variables were pain as measured by the VAS (global assessment of pain, described at the start of this section), the plantar fascia thickness (PFT) in millimetres measured by ultrasonography in the longitudinal plane, and the active extension of the ankle both in passive soleus (AES) and passive gastrocnemius (AEG) as evaluated with the help of a goniometer. The results of this evaluation are presented in Table 2 under the form of $\bar{x}$ ± σ, where $\bar{x}$ is the mean and σ is the standard deviation. 

### 2.5. Statistical Analysis

The descriptive statistical analysis of the variables recorded during the study was made using IBM SPSS v22.0 software. The variables were expressed as mean ($\bar{x}$) and standard deviation (σ). As it was a small sample (*n* < 30), the normality of the variables was checked with the Shapiro–Wilk test. When the variables presented a normal distribution, the Student’s *t*-test was used to compare the measurements; when the distribution was not normal, the comparison was made with the Wilcoxon test. Whenever the results were statistically significant, all necessary tests were made to confirm that the variables were parametric. In all these tests, *p*-values < 0.05 were assumed to be statistically significant with a confidence interval of 95%.

## 3. Results

This section describes the evolution of each of the clinical variables considered in the section ‘Data collection’ during the entire period of the study (6 months). Just as in the previous section, the results are expressed as mean ($\bar{x}$) and standard deviation (σ). 

### 3.1. Evolution of the Clinical Variables between Consecutive Visits

Table 3 shows the evolution (negative values represent a decrease and positive values represent an increase) of the study variables ($\bar{x}$ ± σ) between control visits, as well as the *p*-value that reflects the statistical significance of the evolution. Of the 28 *p*-values presented, 22 are below 0.05, meaning that the evolution of the variables is statistically significant, which supports the effectiveness of the treatment. Non-significant *p*-values in the table have been marked with an asterisk. All these values can be seen from Visit 3 (D30) onwards and are mostly concentrated between Visits 4 (D90) and 5 (D180). This means that while the patients’ condition continues to improve until the 6 month period is over, the most significant improvement takes place in the first weeks. 

**Figure 6:**
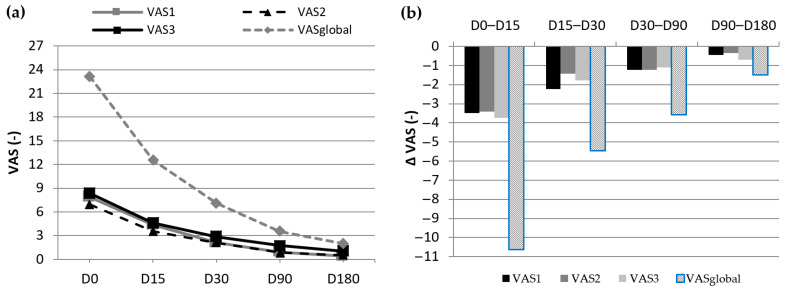
Evolution of the four VAS pain typologies throughout the study.

**Figure 7:**
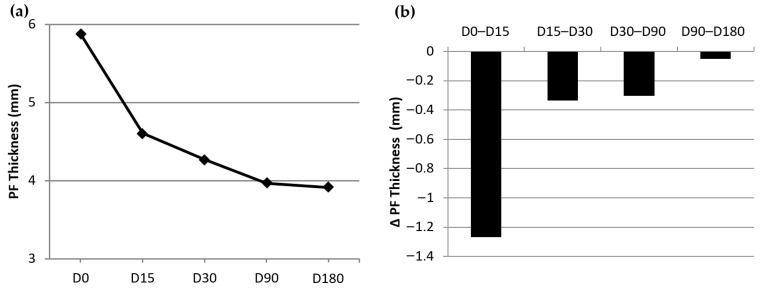
Evolution of the plantar fascia thickness throughout the study.

A series of graphs are presented to give a clearer image of the evolution of the results for each variable. The way the average of the four VAS pain typologies continues to decrease (Figure 6a) clearly reflects a gradual improvement. The evolution of these variables between visits (Figure 6b) shows that the improvement became progressively less marked but continued until the last visit. The same type of evolution (gradual improvement) occurs in the case of plantar fascia thickness (PFT), which is presented in Figure 7. Figure 7a presents the evolution of the decrease in the PFT from D0 to D180, and Figure 7b displays the difference experienced in PFT between visits.

**Figure 8:**
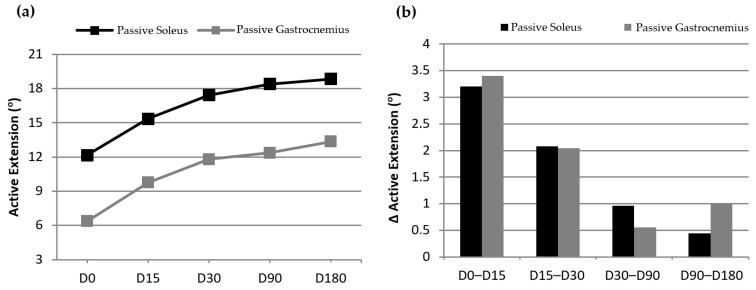
Evolution of ankle mobility throughout the study.

Finally, Figure 8a shows the evolution of the mobility of the ankle (active extension), measured in both passive soleus and passive gastrocnemius. Both variables show that the mobility of the foot affected by PFS increased in each control visit following the infiltration with PRP. The evolution of this variable between visits (Figure 8b) shows that the improvement became progressively less marked but continued until the last visit.

### 3.2. Evolution of Clinical Variables in Relation to the Day of Treatment

Whatever the pathology, it is particularly important to measure the evolution of the patients’ condition compared to the baseline. This period is assumed to correspond to their worst clinical status. Table 4 shows the evolution of all the variables at each of the control visits in relation to the beginning of the treatment (D0) and their respective statistical significance. In this case, all values are statistically significant (*p*-value < 0.05). This confirms the favourable evolution throughout the study of the patients undergoing the treatment described herein.

The mean value of all simple VASx decreased by more than 6 points, while the mean value of VAS_global_ decreased by more than 21 points. The mean value of PFT decreases by 1.96 mm. This reduction is almost twice the size as the one generated by low-voltage electrical impulses [31] (1.05 mm), until now considered one of the most significant in the technical literature. Finally, in what concerns the active extension of the ankle, the mean mobility increases by 6° in the passive soleus and 7° in the passive gastrocnemius. As a final observation of results, it should be mentioned that since the fourth visit (D90), none of the patients have had any clinical symptoms requiring treatment.

## 4. Discussion

The diagnosis of Plantar Fascia Syndrome (PFS) is the result of grouping different symptoms and signs of a multifactorial pathology located in the enthesis between the plantar fascia and the medial calcaneal tuberosity.

Any biomechanical alternation of one element of the ankle’s extensor apparatus (the plantar flexor), which comprises the triceps surae, the calcaneus, and the plantar fascia, could cause PFS. For instance, a contracture of the triceps surae (composed of the gastrocnemius, soleus, and plantaris muscles, converging with the Achilles tendon) due to excessive activity could disrupt the positioning of the calcaneus during the walking movement. This alteration could potentially modify the contraction–distension mechanisms of the fascia, leading to the development of the pathology. However, as there is no real knowledge of the pathogenesis of PFS, nor even of the pathology that occurs in this anatomical area, no consensus has been reached either in terms of definition or in terms of the name to apply to the injuries found in the plantar fascia and in the calcaneus. This makes it easy to think that there is no effective conservative treatment capable of solving this very painful pathology. In addition, due to the lack of rigorous studies worldwide based on scientific evidence, it has not been possible to make categorical statements about the true usefulness of PRP in the treatment of different tendon and aponeurosis diseases, and, more specifically, of the pathology affecting the enthesis between the plantar fascia and the calcaneus. 

The virtues of the technique presented in this paper are based, firstly, on knowing how to define the structures damaged in the PFS, that is the plantar fascia or aponeurosis and the calcaneus; secondly, on the knowledge of the PRP therapeutic capacity both in soft tissue (the fascia) and in hard tissue (the bone); and thirdly, and lastly, on having determined the adequate dosage of PRP. The decision to administer no more than 2 mL of PRP was supported by the anatomical space existent in the hindfoot and by the history of the angiogenic effect of PRP.

The treatment of PFS with one infiltration of 2 mL of PRP has led to clinical improvements that can be labelled as successful when evaluated according to the published criteria [37] because it has been objectively shown that the improvements achieved at Day 180 are much higher than the 25% limit that the scientific literature [37] deems as a minimum for success (>90% in the VAS typologies, >30% in the plantar fascia thickness, and >50% in the active extension of the ankle). 

Another detail that must be taken into consideration is that in this multicentre trial, only one PRP infiltration was used to treat PFS Grade III, and the integrity of the fascia and of the bone was fully respected as opposed to other authors who treated PFS with three infiltrations at intervals of 7 days [38,39,40,41] or two infiltrations at intervals of 7 days [42] or 14 days [43,44]. Other authors claim to have treated chronic PFS with a single infiltration, but the reality is quite different as they have used different points of infiltration in the same session [45]. It is a fact that there are trials based on a single infiltration of PRP. In some of them, a 3 mL dose was used [46,47,48,49,50,51,52,53,54] which is 50% more PRP than the PRP used in the trial (2 mL) presented in this paper. Other authors used larger doses, such as 4 mL of PRP [55] or even 5 mL of PRP in a single infiltration [56], which represents 150% more PRP than used in this trial. Most existing trials infiltrated the PRP using the peppering technique [43,44,48,49,52,53,57,58], which usually generates a cracking sensation in patients that could be painful [59]. The technique presented here is based on a single infiltration with just one redirection, allowing the application of PRP to the entire plantar fascia inserted in the calcaneus, as well as to all the medial tuberosity, which are the anatomical elements requiring treatment. Yet the most significant aspect is the number of extra treatments (listed as complementary treatments) which almost all the authors needed to achieve the remission of the patients’ symptoms. 

Another aspect to stress is that before infiltrating the feet diagnosed with chronic PFS, some authors perform either a posterior tibial nerve block [7,46] or local anaesthesia at the infiltration site [7,47,51,57,58] and this is a totally different procedure. In this trial, there was no nerve block or anaesthesia, and the infiltration area was only sprayed with chloroethyl Chemirosa with the exclusive purpose of reducing skin sensitivity at the time of the puncture. 

This trial is also particular with regard to the concerns of the diagnosis and the control of the evolution of the clinical variables used. There are no known trials that have assessed four different pain typologies with the 10 points Visual Analogue Scale nor any studies reflecting the results of the active extension of the ankle in passive soleus and in passive gastrocnemius in patients treated with PRP. However, there are studies like this one that reflect the decreasing values of PFT in patients treated with PRP [48,51,53,54,55,60].

Due to the fact that the therapy is based on etiopathogeny and is not based on reducing the symptoms of the injury, the effect of the infiltration is long. The success of the therapeutic procedure with PRP lies in its regenerative effect on soft tissue (plantar fascia) and hard tissue (calcaneus), which recover their prepathologic state. 

Here, it is also important to remember that none of the so-called complementary treatments were used, so it can be stated that all the curative effect lies in the PRP and in the administration technique herein described. Moreover, using only 2 mL of PRP (a very small quantity when compared with the above-mentioned treatments) it is possible to remark the therapeutic power of PRP.

Finally, it is important to point out the two most significant limitations of the study. The study sample is small, although it exceeds the minimum size (24 patients) calculated through a pilot study of 10 subjects. It should be considered that the study did not receive financial support, and the price of the material used was high, so the number of patients was optimized and adjusted as much as possible to the minimum number of patients to guarantee the representativeness of the sample. It is also important to mention that other studies have similar samples of patients [40,41,48,52,53,55] treated with PRP. Likewise, according to the authors’ criteria, the 25 patients under study rigorously represent the characteristics of the standard type of patient who attends clinical practice since they were recruited in the two study centres by highly qualified professionals, minimizing possible bias. The second important limitation is the nonexistence of a control group. In this case, the lack of a control group is justified for two reasons. It is very difficult to inject a placebo product in the enthesis between the plantar fascia and calcaneus without causing harm to the patients. Furthermore, some authors [61,62] consider that these types of studies without control groups are still valid for evaluating new treatments if they rely on before–after, implicit, or historical comparisons as a proxy for an ideal comparison group. It means that in these studies, a variable of interest is measured before and after an intervention in the same participants, as it is performed in this paper. The basis for deriving a conclusion from these studies is the temporal relationship of the measurements to the intervention. However, it is true that the outcome can instead be related to other changes that occurred around the same time as the intervention. The bias that the study could present due to the nonexistence of a control group has been tried to be minimized by including only patients diagnosed with recalcitrant PFS lasting for more than 12 months and using only one analysis variable that could be considered subjective, the VAS_global_, and three objective analytical variables: PFT, AES and AEG.

## 5. Conclusions

The main conclusions that can be drawn from this paper are as follows:

1. One single infiltration with 2 mL of platelet-rich plasma (PRP) in patients diagnosed with the Plantar Fascia Syndrome (PFS) is effective in the treatment of this pathology. Healing times are shortened and the effect is maintained in the long term.
2. There is a significant decrease in the pain measured during the 6 month study with the Visual Analogue Scale (first steps in the morning, first steps after a long rest, and sensitivity to touch in the affected area) according to the protocol schedule (Days 15, 30, 90, and 180).
3. There is a significant decrease in the plantar fascia thickness (PFT) measured during the 6 months study using ultrasonography according to the protocol schedule.
4. There is a significant increase in the active extension of the ankle as measured during the 6 month study, both in passive soleus and passive gastrocnemius according to the protocol schedule.
5. The improvement in clinical variables was maintained throughout the 6 months of the study.
6. After 6 months, there is a clear correlation between plantar fascia thickness decrease and pain decrease.

## Figures and Tables

**Table 1:** Demographic features.

	Age (Years)	Weight (kg)	Height (m)	BMI (kg/m^2^)	Symp. Dura. (Months)
**Mean ± σ**	48.9 ± 9.74	76.44 ± 15.10	1.64 ± 0.09	28.24 ± 4.65	15.76 ± 11.91

BMI: Body Mass Index; Symp. Dura.: Duration of symptoms.

**Table 2:** Data was collected during the control visits.

	D0	D15	D30	D90	D180
**VAS_1_**	7.86 ± 1.59	4.38 ± 2.08	2.14 ± 2.10	0.9 ± 1.44	0.44 ± 0.82
**VAS_2_**	6.98 ± 1.49	3.54 ± 2.37	2.1 ± 2.08	0.86 ± 1.32	0.52 ± 0.82
**VAS_3_**	8.36 ± 1.38	4.62 ± 2.59	2.84 ± 2.09	1.74 ± 1.76	1.04 ± 1.39
**VAS_global_**	23.18 ± 3.58	12.54 ± 6.33	7.08 ± 5.71	3.5 ± 3.52	2 ± 2.58
**PFT**	5.87 ± 1.04	4.60 ± 1.47	4.27 ± 1.42	3.96 ± 1.30	3.91 ± 1.24
**AES**	12.16 ± 4.97	15.36 ± 4.5	17.44 ± 4.23	18.4 ± 4.25	18.84 ± 3.99
**AEG**	6.36 ± 5.84	9.76 ± 4.98	11.8 ± 3.98	12.36 ± 3.91	13.36 ± 3.55

**Table 3:** Evolution of the values in relation to the previous visit.

	D0–D15	*p*-Value	D15–D30	*p*-Value	D30–D90	*p*-Value	D90–D180	*p*-Value
**VAS_1_**	−5.72 ± 2.24	0.000	−2.24 ± 1.73	0.000	−1.24 ± 1.76	0.003	−0.46 ± 1.49	0.138 *
**VAS_2_**	−4.86 ± 1.74	0.000	−1.44 ± 1.85	0.000	−1.24 ± 2.34	0.017	−0.34 ± 1.04	0.117 *
**VAS_3_**	−5.52 ± 2.31	0.000	−1.78 ± 2.58	0.008	−1.10 ± 2.25	0.030	−0.7 ± 1.19	0.007
**VAS_global_**	−16.10 ± 5.42	0.000	−5.46 ± 4.89	0.000	−3.58 ± 5.47	0.007	−1.5 ± 2.90	0.016
**PFT**	−1.60 ± 1.13	0.000	−0.33 ± 0.61	0.000	−0.30 ± 0.41	0.002	−0.05 ± 0.320	0.424 *
**AES**	5.28 ± 3.51	0.000	2.08 ± 3.93	0.017	0.96 ± 4.39	0.245 *	0.44 ± 4.33	0.530 *
**AEG**	5.44 ± 4.95	0.000	2.04 ± 3.67	0.019	0.56 ± 4.16	0.384 *	1.56 ± 3.41	0.034

* *p*-value not statistically significant with a confidence interval of 95%.

**Table 4:** Evolution of the variables in relation to the start of the treatment (D0).

	D0–D15	*p*-Value	D0–D30	*p*-Value	D0–D90	*p*-Value	D0–D180	*p*-Value
**VAS_1_**	−3.48 ± 1.94	0.000	−5.72 ± 2.24	0.000	−6.96 ± 1.91	0.000	−7.42 ± 1.93	0.000
**VAS_2_**	−3.42 ± 2.20	0.000	−4.86 ± 1.74	0.000	−6.10 ± 2.06	0.000	−6.44 ± 1.68	0.000
**VAS_3_**	−3.74 ± 2.70	0.000	−5.52 ± 2.31	0.000	−6.62 ± 2.22	0.000	−7.32 ± 1.82	0.000
**VAS_global_**	−10.64 ± 6.35	0.000	−16.10 ± 5.42	0.000	−19.68 ± 5.18	0.000	−21.18 ± 4.65	0.000
**PFT**	−1.27 ± 1.01	0.000	−1.60 ± 1.13	0.000	−1.91 ± 1.07	0.000	−1.96 ± 1.09	0.000
**AES**	3.20 ± 4.42	0.000	5.28 ± 3.51	0.000	4.97 ± 4.25	0.000	6.68 ± 5.28	0.000
**AEG**	3.40 ± 4.95	0.000	5.44 ± 4.95	0.000	5.85 ± 3.91	0.000	7.00 ± 5.22	0.000

## Data Availability

Data is available upon reasonable request.
